# The therapeutic efficacy of guided therapy for PCI after acute myocardial infarction: A meta-analysis

**DOI:** 10.1097/MD.0000000000036183

**Published:** 2023-11-24

**Authors:** Meixia Sun, Yi Ding, Kang Chen, Yanwen He, Yukun Zhang, Yue Zhuo, He Zhuang

**Affiliations:** a School of Rehabilitation Medicine, Shandong University of Traditional Chinese Medicine, Jinan, Shandong, China; b The Second Affiliated Hospital of Shandong University of Traditional Chinese Medicine, Jinan, Shandong, China.

**Keywords:** acute myocardial infarction, lead therapy, meta-analysis, percutaneous coronary intervention

## Abstract

**Objective:**

To systematically evaluate the effects of lead therapies on percutaneous coronary intervention (PCI) after acute myocardial infarction (AMI).

**Methods:**

A randomized controlled trial (RCT) in the CNKI, Wanfang, VIP, ProQuest, PubMed, Cochrane Library, Scopus, and Web of Science databases was searched until January 2023. Two researchers strictly screened and checked the included literature, extracted relevant data, and used the Cochrane Manual to assess the risk quality of the literature. Using RevMan 5.3 software, Meta-analysis of 4 main outcome measures [cardiac function-related indicators, 6-minute walking distance (6 MWT), quality of life (SF-36), Seattle angina pectoris scale (SAQ)], and 3 secondary outcome measures [adverse event incidence, death incidence, and readmission rate].

**Results:**

22 studies were finally included with 1754 subjects, but the overall quality of the included studies was not high. The results of the meta-analysis showed that, in the cardiac function-related indicators compared to controls, improved left ventricular ejection fraction (LVEF) index (MD = 1.42, 95%CI [−0.94, 3.79], *P* < .00001); however, compared with the Baduanjin group, Tai Chi ball + Baduanjin group and control group, there was no significant difference (*P* > .05); compared with the control group, the guidance therapy group improved the left ventricular end-diastolic volume (LVEDV) index (MD = −4.67, 95%CI [−6.8, −2.71], *P* < .00001). In comparison, the lead group improved the 6 MWT (MD = 69.44, 95%CI [30.12, 108.76], *P* < .00001); the SF-36 score (MD = 10.05, 95%CI [8.68, 11.42], *P* < .00001])and the SAQ score (MD = 6.2, 95%CI [3.97, 8.44], *P* < .00001). Among the secondary outcome measures, the incidence of adverse events was statistically significant (RR = 0.17, 95%CI [0.1, 0.32], *P* < .00001); statistically significant (RR = 0.29, 95%CI (0.1, 0.87), *P* < .00001); readmission (RR = 0.39, 95%CI [0.17, 0.87, 0.89], *P* < .00001).

**Conclusion:**

Based on the current study, combining conventional therapy/ exercise or using simple lead therapy after PCI can improve the treatment effect and improve the quality of life.

## 1. Introduction

Acute myocardial infarction is usually caused by blockage of heart vessels, myocardial ischemia, hypoxia and necrosis, myocardial damage, and cardiac dysfunction,^[1,2]^ The clinical manifestations of acute myocardial infarction include dyspnea, persistent severe retrosternal pain, and chest pain, which seriously affect the quality of life of patients.^[3]^ With the rapid development of society, people lifestyles and habits have changed, and life pressure is also gradually increasing. Coupled with the accelerated aging of the population, the incidence of acute myocardial infarction in China is increasing year by year. Percutaneous coronary intervention (PCI) is still the preferred treatment option for acute myocardial infarction, which can effectively reduce the mortality rate of patients^[4,5]^ In addition. Early postoperative rehabilitation and continuous secondary prevention are also key to improving patient outcomes. Exercise rehabilitation, an important component of cardiac rehabilitation, can significantly improve cardiac function and quality of life. Traditional Chinese medicine guidance therapy, such as Taijiquan and Baduanjin, is the physical exercise therapy combining “form,” “qi” and “god.” Adjusting the Yin and Yang of the human body plays the role of supporting healthy and dispelling evil^[6–7]^ At the same time, it can also reduce the risk factors, improve disease safety, and improve the quality of life of patients.^[8]^ The guiding treatment takes the body, interest and heart as the basic elements, pays attention to the combination of physical movement, breathing and psychological adjustment, and assists the internal adjustment of drugs, so that the body achieves the state of “Yin and Yang secret^.^”^[9]^ At present, there are many guidance therapies are currently being used for rehabilitation treatment after PCI for acute myocardial infarction. However, there is a lack of evaluation of the impact of different guidance therapies on the efficacy and quality of life after PCI for acute myocardial infarction; therefore, the selection of appropriate guidance therapy has high research value and clinical significance for the improvement of PCI after acute myocardial infarction. This study used a meta-analysis method to evaluate the efficacy and quality of life of guided therapy for PCI for acute myocardial infarction in order to provide a clinical basis for lead therapy in clinical treatment and rehabilitation of PCI for acute myocardial infarction.

## 2. Data and methods

The search strategy was to search the full text databases of Chinese academic journals (CNKI), Wanfang database (Wanfang), VIP database (VIP), China Biomedical database (SinoMed), PubMed, Cochrane Library, Embase, Web of Science, and 8 Chinese and English databases. The search will be completed until January 2023, and the languages will be Chinese and English. The Chinese search terms were: “Baduanjin, Wuqinxi, 6-character formula, Tai Chi exercise, Tai Chi, Tai Chi ball, Yi jin Jing, Qigong, practice, guidance therapy” acute myocardial infarction “and” percutaneous coronary intervention “; The English search term is: ““ Baduanjin,” Wuqinxi, 6-character formula, Tai Chi exercise, Taijiquan, Taiji ball, Yi Jin Jing, Qigong method, practice method, guidance therapy “acute myocardial infarction” and “percutaneous coronary intervention” uses the joint retrieval method of subject words plus free words.

Literature inclusion and exclusion criteria 2.2.1 inclusion criteria: Study type: randomized controlled trials (RCTs). Subjects who met the diagnostic criteria for acute myocardial infarction [Cai Yu] and underwent PCI surgery. Interventions: The control group adopted conventional therapy, conventional treatment, traditional medicine therapy, other rehabilitation therapy, routine exercise, and routine care; the guidance group adopted simple guidance therapy, or combined guidance therapy based on the control group, which included Baduanjin, Wuqinxi, 6-character formula, Tai Chi exercise, Taijiquan, Tai Chi ball, and Yi Jin Jing. If multiple group comparisons occurred in the collected literature, only the groups related to this study were selected for inclusion. Outcome measures:Primary outcome measures: cardiac function, 6MWT, SF-36, SAQ, and 3 secondary outcome measures: incidence of adverse events, incidence of death, and readmission.

Exclusion criteria repeatedly published, data errors; only added Baduanjin exercise intervention between the 2 groups; cases were CHF disease with other organic diseases; animal experiment, review, experience summary; could not find full text; experiment design without comparable baseline data between groups; plagiarism and other problems; and does not involve a selected outcome indicator.

### 2.1. Literature screening and inclusion

By reading the title and abstract, we screened the documents out of the above inclusion criteria, then read the full text, and the documents of all the documents were judged whether to be included in the paper, the opinions of the third-party researchers.

### 2.2. Data extraction

Data were extracted from the original article by 2 independent researchers. Disagreements were resolved by a third researcher. The inclusion criteria were as follows: author, year, sample size, intervention, course of treatment, main evaluation indicators, and adverse reactions.

### 2.3. Risk of bias assessment

The quality of each trial was independently assessed by 2 researchers according to the Cochrane Risk of Bias instrument. Each item was classified as high-, uncertain-, or low-risk. Any disagreements were resolved by a third independent researcher.

### 2.4. Statistical analysis

The Meta-analysis was performed using RevMan 5.4 software. For the included literature with insufficient information, we contacted the authors by email to the requested data as far as possible. For continuously calculated data (different WOMAC dimensions and VAS), mean difference (MD) and 95% confidence intervals (Cis) were calculated. The hazard ratio (RR) and 95% confidence interval (CI) were used as dichotomous results (response rates). According to the availability of data in this study, the subgroups of different intervention types were analyzed in the control group. I^2^ Tests were used to measure the statistical heterogeneity when I^2^ < 40% and *P* > .1; otherwise, the random effects model was used. Sensitivity analyses were performed based on the trial quality, sample size, intervention duration, intervention session duration, and intervention frequency. A funnel plot was used to assess the potential publication bias.

## 3. Results

### 3.1. Literature search results

**Figure 1.**
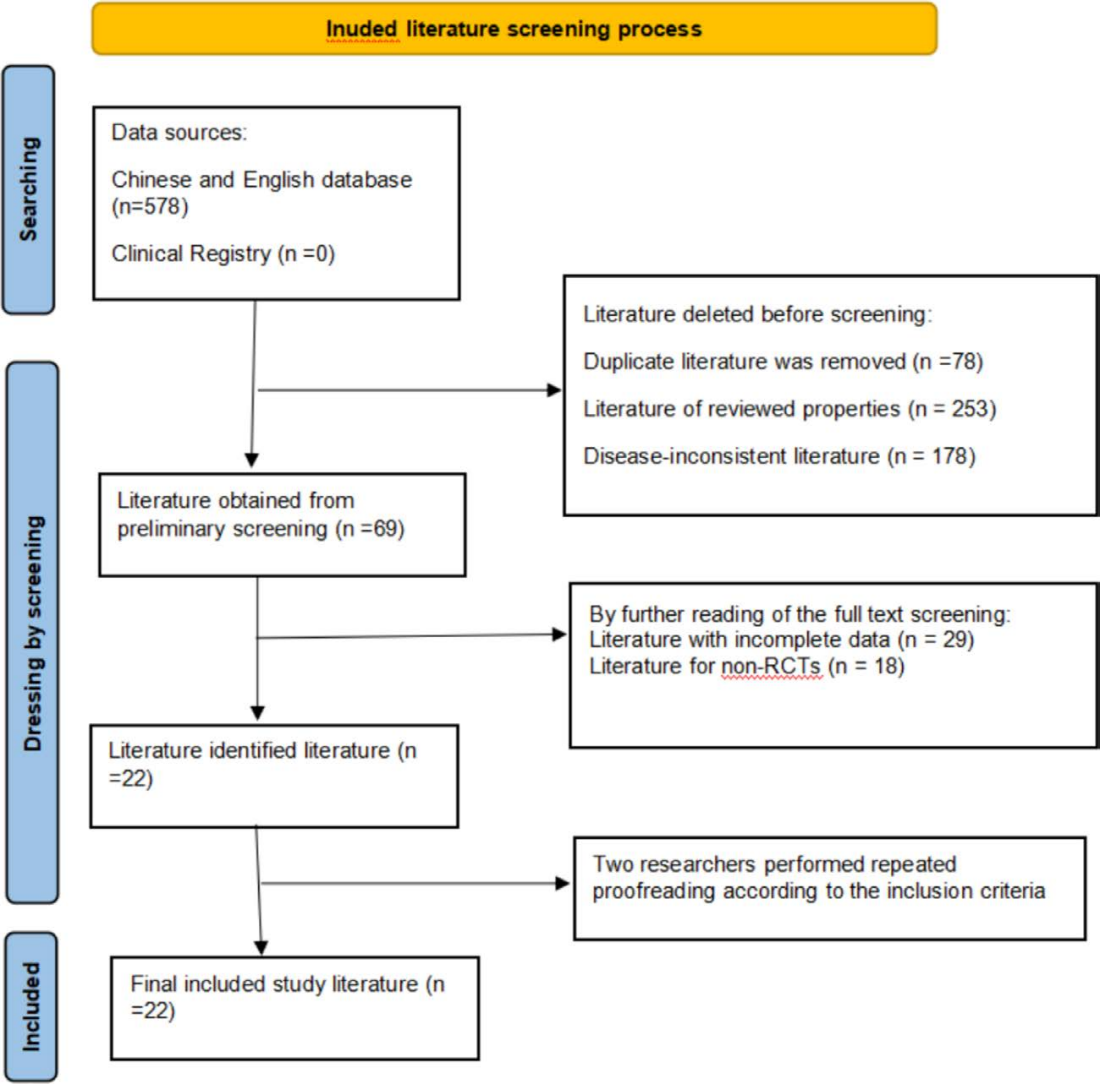
Results of the literature search.

A total of 578 articles were retrieved in the above Chinese and English databases, including 578 Chinese articles and 0 English articles.0 studies in the clinical registry. The screen was conducted according to other inclusion exclusion criteria, and 22^[10–31]^ were included, as shown in Figure 1.

### 3.2. Basic characteristics of the included studies

A total of 1 261 study subjects were included, Among these patients, 631 cases were included in the lead group, 630 cases in the control group; The sample size of a single study was from 40 to 300 cases; The control group included conventional therapy, conventional therapy + traditional Chinese medicine therapy, conventional therapy + other rehabilitation therapy, usual exercise, usual care, The lead group was TCM lead therapy, or combined lead therapy in the control group, Guiding therapy includes Baduanjin (13), Tai Chi (2), Tai Chi ball (1), Tai Chi sports combined with Baduanjin (1) and Tai Chi ball combined with Baduanjin (2); In 15 studies using cardiac function as an outcome measure, Eight studies with 6 MWT as an outcome measure, Seven studies with SF-36 as the outcome measure, There were 2 studies using ADL as the outcome measure. See Table 1.

**Table 1:** Presents their basic characteristics.

Study ID	Sample capacity (T/C)	Intervention study		Course of treatment	Observational indicators	Untoward effect
		T	C			
Na Wei^[10]^ 2023	60 (30/30)	Routine rehabilitation and nursing + Baduanjin	Routine rehabilitation care	4 wk	Readmission rate, incidence of major adverse cardiovascular events and post-care cardiac function indicators, ADL	One patient was included in the observation group and the control group 6
GuoGuo Liu^[11]^ 2023	60 (30/30)	Yixin drink + badbrocade	Daily activities	12 wk	Balance index, fall risk index, cardiopulmonary function index, and quality of life	Not described
XueFei Liang^[12]^ 2022	48 (24/24)	Routine treatment + Baduanjin	Conventional therapy	6 mo	SF-36, integrated quality of survival scale for CHD	Not described
Jing Zhou^[13]^2022	100 (50/50)	Conventional treatment + Baduanjin + blood house blood soup	Conventional treatment plus bedside activities	24 wk	LVEF, NT-proBNP levels, and 6 MWT	Not described
Yu Cai^[14]^ 2022	90 (45/45)	Regular exercise instruction + badbrocade	Routine exercise instruction	2 wk	METmax, 6MWT, PSQI , VO2max	Not described
GuoGuo Liu^[15]^ 2021	60 (30/30)	Routine treatment + Baduanjin	Conventional therapy	12 wk	NT-proBNP, LVEF, 6 MWT, SAQ, QOL-B R EF, episodes of angina pectoris, restarting acute myocardial infarction, target vessel revascularization, deterioration of cardiac function, and number of cardiac deaths	Twelve patients were included in the observation group and 28 patients were included in the control group
Liang Kang^[16]^ 2021	60 (30/30)	Routine drug therapy + Baduanjin exercise group	Conventional medication	In February	Cardiac function indicators, LDL-C, TC and other biochemical indicators 6 MWT, Mets value, cardiopulmonary function indicators, heart rate variability index; SAQ, TCM syndrome integral scale and SF-36, Hamilton Anxiety and Depression Scale	Do not appear
JuanXue Wang^[17]^ 2021	80 (40/40)	Routine exercise guidance + Baduanjin therapy	Routine exercise instruction	2 wk	SF-36, ADL	Not described
Li Su^[18]^2021	60 (30/30)	Routine treatment + Baduanjin	Conventional therapy	12 wk	LDH, H-FABP, NO, IFN- γ, IL-10, IcT nI, NT-proBNP, MPO, NOS, vWF, LVESD, LVEF, LVEDD, 6 MWT, ADL, and clinical efficacy	Do not appear
WuMei Xia^[19]^2021	90 (45/45)	Routine treatment + Tai Chi	Conventional therapy	8 wk	Cardiac function, metabolic indicators, inflammation, occurrence of adverse cardiac events	One patient was included in the observation group and 8 patients were included in the control group
Xu Guo^[20]^ 2019	120 (60/60)	Conventional treatment plus Baduanjin combined with Baduanjin	Conventional therapy	1 mo	NT-proB B NP, LVEDV, LVEF, 6 MWT, clinical symptom integral, HAMA	Not described
JiaMei Wang^[21]^ 2018	150 (75/75)	Conventional treatment + sitting type Baduanjin	Conventional therapy	6 mo	LVEF, LVESD, LVEDD, BNP, 6 MWT, readmission rate, and mortality rate	Do not appear
GuoBin Li ^[22]^ 2019	60 (30/30)	Conventional treatment + sitting tai Chi ball, Baduanjin sports	Conventional rehabilitation +	2 mo	NT-proBNP, LVEF, LVEDV, TCM symptom integral, SAQ, Hamilton Anxiety Scale integral, Hamilton Depression Scale integral, Mets without oxygen threshold, VE without oxygen threshold, kg oxygen intake at no oxygen threshold	One patient experienced dizziness and discomfort after the cardiopulmonary exercise test with no special treatment
XiaoDuo Zhang^[23]^ 2018	60 (30/30)	Conventional treatment plus Baduanjin combined with tai Chi ball	Conventional therapy	1 mo	NT-proBNP, LVEF, LVEDV, SAQ, MetS, and clinical symptoms.	Two patients showed slight arm weakness after exercise and no special treatment
HaiYang Shi^[24]^ 2023	58 (31/27)	Conventional treatment + Taijiquan combined with acupuncture treatment	Conventional therapy	12 wk	HRV, cardiac function, 6 MWT, and SF-36	Five patients were in the Tai Chi group and 11 patients were in the control group
DongMei Yu^[25]^2021	64 (32/32)	Routine treatment + Tai Chi	Conventional therapy	4 mo	Cardiac function status, 6 MWT, TUCT, NT-proBNP, and cardiac color ultrasound	There were 5 patients in the test group and 11 patients in the control group
Li Lu^[26]^ 2020	80 (40/40)	Conventional treatment + heart pulse warm Yang through collaterals paste with tai Chi ball fitness method	Conventional therapy	1 mo	Clinical symptoms, improvement in cardiac function, anxiety, depression score, and quality of life score	Not described
Mei Lu^[27]^2022	96 (48/48)	Routine treatment + Tai Chi exercise and badJin	Conventional therapy	3 mo	Cardiac function, exercise endurance, quality of life, and the incidence of adverse cardiovascular events	Two patients were included in the test group and 9 patients were included in the control group
Ming Li^[28]^2021	80 (40/40)	Conventional treatment plus Baduanjin combined with auricular acupoint pressure beans	Conventional treatment regimen	3 mo	Echocardiography, 6 MWT, SAQ, clinical symptoms, HAM-A, HAM-D, readmission rate, and mortality status.	Not described
YinHe Cai^[29]^ 2022	60 (30/30)	Routine treatment + Baduanjin	Conventional therapy	3 mo	Heart structural and functional indicators, EQ-5D, and serum inflammatory factors	Do not appear
XueYing Han^[30]^2023	118 (57/61)	Conventional therapy + Taiji ball combined with badJin	Conventional therapy	1 mo	Ability of daily living activities and quality of life	Not described
Shuai Zong^[31]^ 2022	100 (50/50)	Conventional therapy + cardiac rehabilitation exercise combined with Baduanjin	Conventional therapy + cardiac rehabilitation exercise	2 wk	Improvement in cardiac function, exercise endurance and quality of life, and incidence of adverse events	There were 8 patients in the control group and 2 patients in the observation group

ADL = daily life energy scale, AT = anaerobic threshold, BNP = plasma type B brain natriuretic peptide, HAMA = The Hamilton Depression Scale, H-FABP = heart-type fatty acid-binding protein, HRV = heart rate variability, LDH = lactate dehydrogenase in the 2 groups, LVEDD = left ventricular, LVEF = left ventricular ejection fraction, LVESD = LV end-systolic internal diameter, METmax = maximum metabolic equivalent, MPO = myeloperoxidase, NO = nitric oxide, NOS = nitric oxide synthase, NT-proBNP = the level of the N-terminal type-B natriuretic peptide precursor, Peak METs = peak metabolic equivalent, PSQI = the Pittsburgh Sleep Quality Index, QOL-B R EF = quality of life integral, 6 MWT = 6 minutes walking distance, SAQ = Seattle Anginal Pain Scale score, SF-36 = quality of life brief table, TUGT = stand up-walking timing test, IFN-γ = interferon-γ, IL-10 = interleukin-10, IcT nI = myocardial troponin, VO2max = maximum oxygen uptake, VWF = von Willebrand factor.

### 3.3. Offset risk assessment

**Figure 2.**
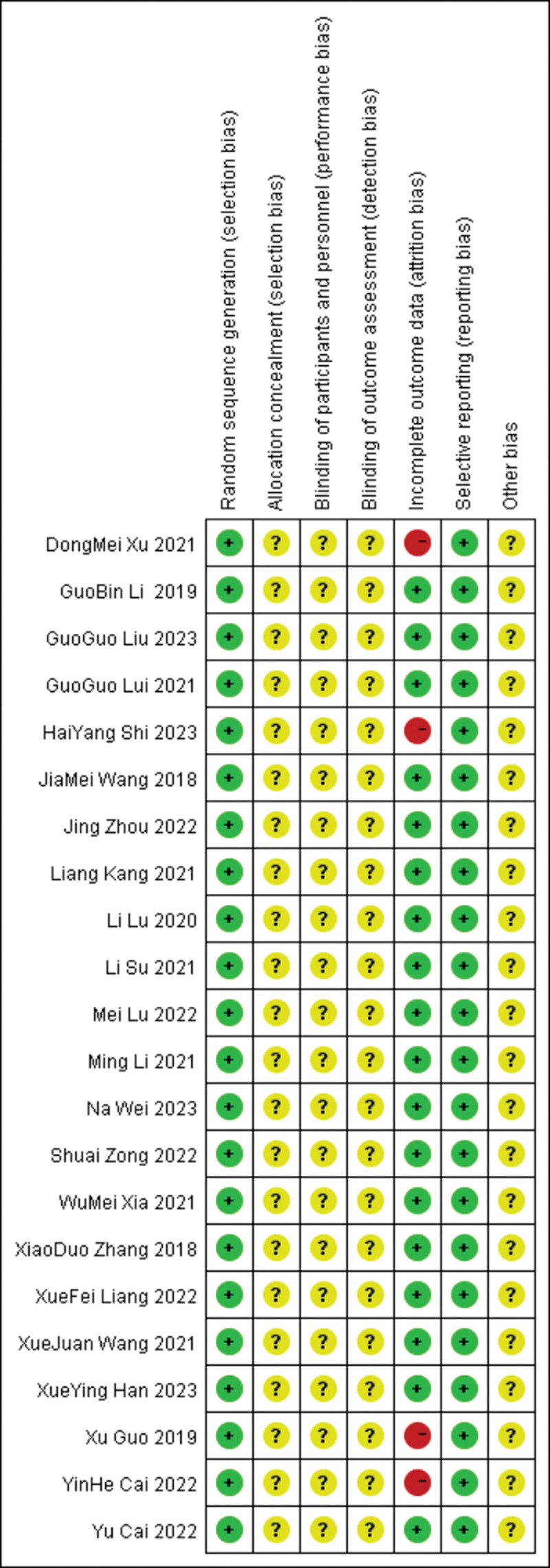
Offset risk assessment. +: Low risk; -: high risk; ?: NK.

12 studies stated the allocation method in the random allocation, and the assessment was low risk, while the remaining trial assessment was unclear; none mentioned the allocation hidden assessment as unclear, the blind method still mentioned no assessment as unclear, 6 trials had shedding cases, and the integrity of the data was high risk; and selective reporting and other offset assessments were low risk. See Figure 2 for further details.

### 3.4. Meta-analysis results

#### 3.4.1. Index related to cardiac function.

##### 3.4.1.1. LVEF.

17 studies ^[10,13,15–16,18–29,31]^ reported LVEF, including 9 in the Baduanjin group, 4 in the Tai Chi ball + Baduanjin group, 3 in the Tai Chi group, and 1 in the Tai Chi ball group. Subgroup analyses were performed according to the different interventions. The Baduanjin group was heterogeneous (*P* < .00001, I^2^ = 92%). Meta-analysis was conducted using the random-effects model, with no significant difference, indicating the LVEF index of patients in the Baduanjin group. [MD ＝ 0.62, 95%C (−2.17, 3.41), *P* = .66].

Due to the large heterogeneity of this index, sensitivity analysis is needed, and the original forest map showed that the publication bias of 5 articles^[13,14,16,18,22]^ was large, and the heterogeneity was significantly reduced after elimination (*P* = .11 and I^2^ = 48%). A fixed-effects model can be used. The results still showed that the LVEF index of patients with improvement (MD = 4.16,95%CI [2.9, 5.42], *P* < .00001) had low sensitivity and relatively stable results.

Heterogeneity was tested in the Taiji ball + Baduanjin group, and the results showed heterogeneity (*P* < .00001, I^2^ = 95%). In a Meta-analysis using a random effects model, the difference was not statistically significant, indicating that the Taiji ball + Baduanjin group had an improved LVEF index. [MD ＝ −1.27, 95%C (−7.61, 5.06), *P* = .69].

Due to the large heterogeneity of this index, sensitivity analysis was needed, and the original forest map showed that the publication bias of 2 articles^[23,27]^ was large, and the heterogeneity was significantly reduced after elimination (*P* = .79 and I^2^ = 0%). The fixed-effects model was used for analysis. The results still showed that the taiji ball + Baduanjin group improved the LVEF index (MD = 3.06, 95%CI [1.58, 4.54], *P* < .0001), with low sensitivity and relatively stable results.

The heterogeneity test of Taijiquan showed heterogeneity (*P* < .00001, I^2^ = 98%). Meta-analysis was performed using the random-effect model, which was not statistically significant, indicating the LVEF index of patients. [MD ＝ 1.86, 95%C (−5.04, 8.77), *P* = .6].

**Figure 3.**
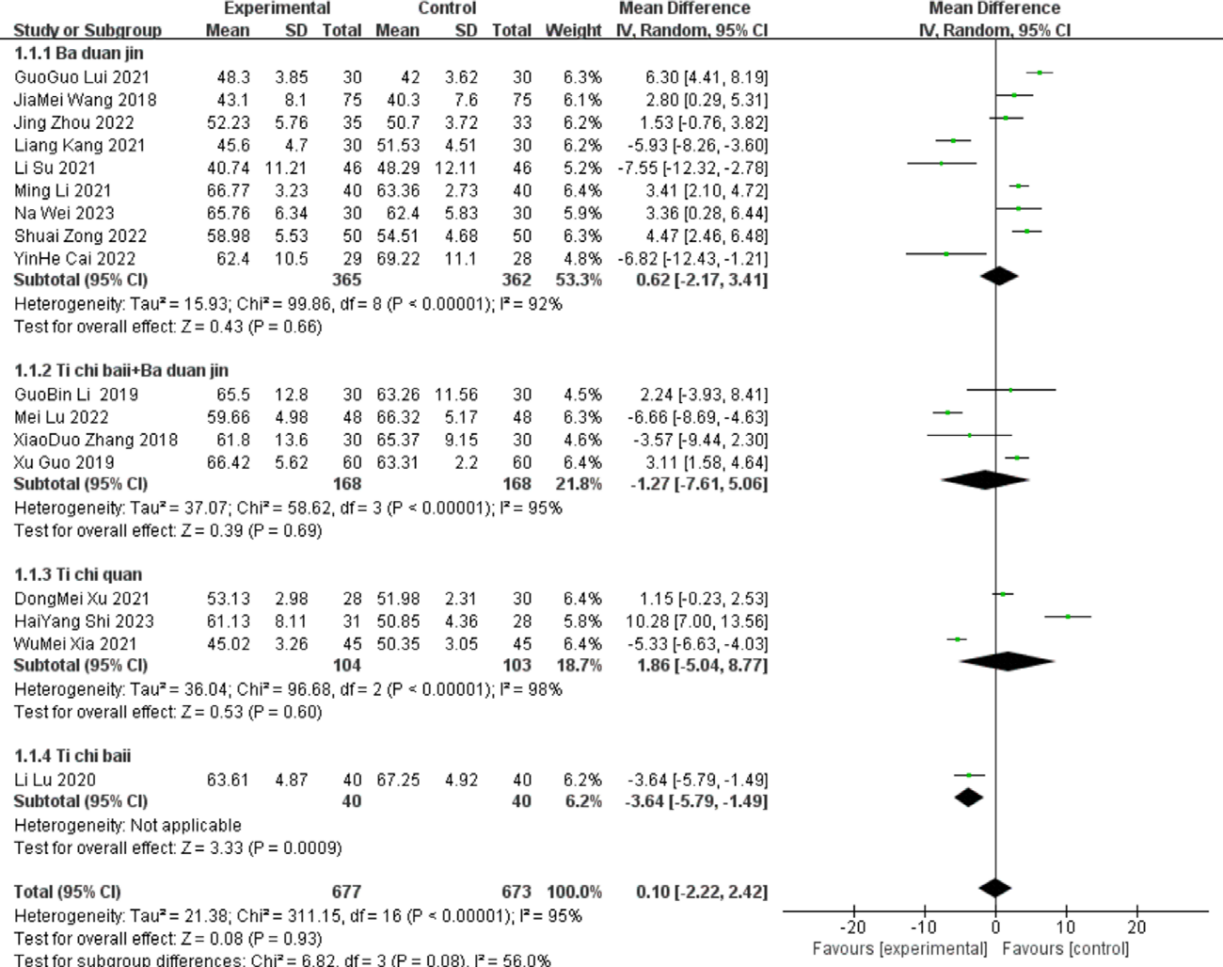
The meta-analysis results of group LVEF comparisons. LVEF = left ventricular ejection fraction.

**Figure 4.**
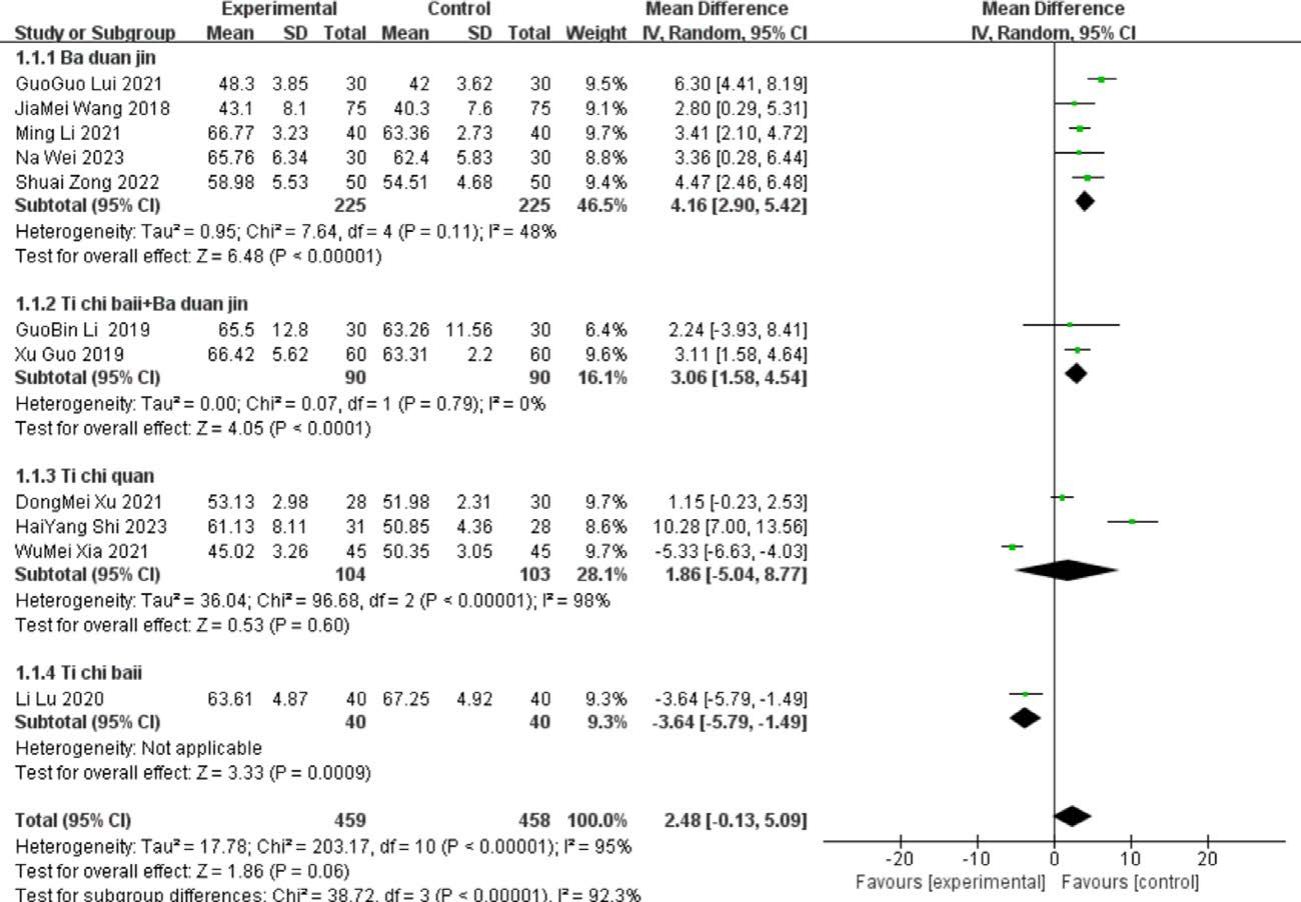
The meta-analysis results of the 2-group LVEF comparisons after sensitivity analysis. LVEF = left ventricular ejection fraction.

As Tai Chi only combined the effect sizes of the 3 studies, the deletion heterogeneity did not change significantly. The^[25]^ course of Yu Dongmei was 4 months, the^[24]^ course of Shi Haiyang^[24]^ was 12 weeks, and the^[18]^ course of Xia Fumei was 8 weeks. The difference in treatment course may be the source of heterogeneity. Only one study was included in the Taiji ball group; therefore, a heterogeneity test was not performed. A Meta-analysis using a random effects model for the included 17 studies showed that the lead group improved the patient LVEF index compared with the control group.[MD = 1.42, 95%CI (−0.94, 3.79), *P* < .00001], shown in Figures 3 and 4.

##### 3.4.1.2. LVEDV.

Seven studies^[10,20,22,23,26,28,29]^ reported LVEDV, including 3 in the Baduanjin group, 3 in the Tai Chi ball + Baduanjin group, and 1 in the Tai Chi ball group. Subgroup analyses were performed according to the different interventions. A heterogeneity test was conducted on the Taiji ball + Baduanjin group, and the results showed good homogeneity (*P* = .56, I^2^ = 0%). Meta-analysis was conducted using the random-effect model, and the 2 groups were statistically significant, indicating that the Taiji ball + Baduanjin group had an improved LVEDV index. [MD ＝ −3.83, 95%C (−4.99, −2.66), *P* < .00001].

**Figure 5.**
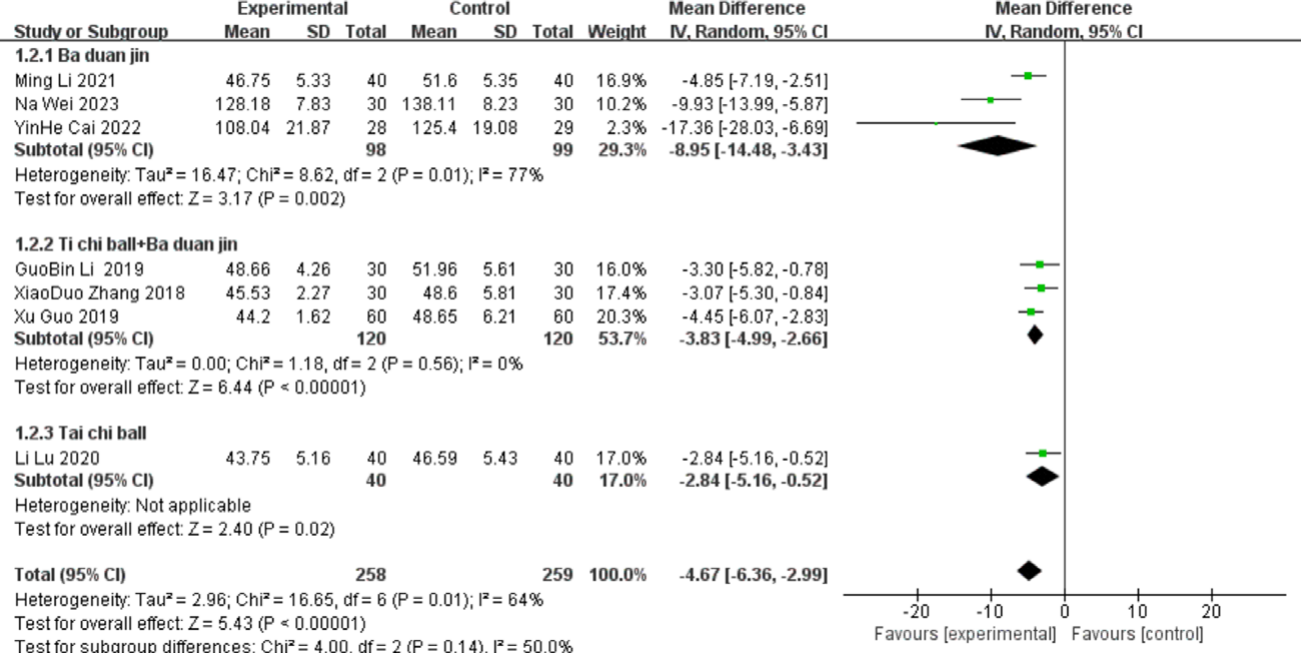
The meta-analysis results of group LVEDV comparisons. LVEDV = left ventricular end-diastolic volume.

**Figure 6.**
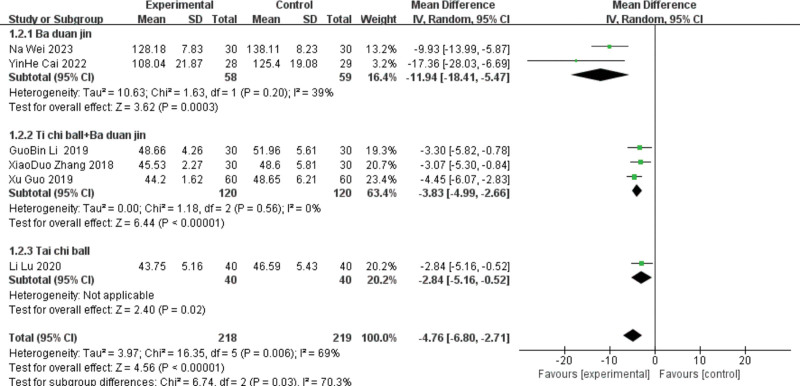
The meta-analysis results of the 2-group LVEDV comparisons after sensitivity analysis. LVEDV = left ventricular end-diastolic volume.

The group were heterogeneous (*P* = .01, I^2^ = 77%). The meta-analysis using the random-effects model was statistically significant, indicating that the group improved the LVEF index. [MD ＝ −8.95, 95%C (−14.48, −3.43), *P* = .002]. Due to the large heterogeneity of this index, sensitivity analysis is required, and the heterogeneity of the study excluding Li Ming [28] (*P* = .2, I = I^2^ = 39%) can be analyzed by the fixed effect model, and the results still showed that lead therapy could improve the LVEDV level [MD =  −11.94, 95%CI (−18.41, −5.47), *P* = .0003]. The results were unchanged, with low sensitivity and stability. Only one study was included in the Taiji ball group; therefore, a heterogeneity test was not performed. Meta-analysis using the random effects model showed that the lead group had an improved LVEDV index compared with the control group (MD = −4.67, 95%CI [−6.8, −2.71], *P* < .000 01). See Figures 5 and 6 for details.

#### 3.4.2. 6MWT.

Ten studies^[13–16,18–21,24–25]^reported 6 MWT results, including 6 in the Baduanjin group, 1 in the Tai Chi ball + Baduanjin group, and 3 in the Tai Chi group. Subgroup analyses were performed according to the different interventions. The Baduanjin group showed heterogeneity (*P* < .00001, I^2^ = 99%). A meta-analysis was performed, and the difference between the 2 groups was statistically significant, indicating that the patients had a 6 MWT index. [MD ＝ 69.5, 95%C (22.37, 116.64), *P* = .004].

Due to the large heterogeneity of this index, sensitivity analysis is required. Excluding 4 articles^[13–15,21]^ significantly decreased heterogeneity (*P* = .45 and I^2^ = 0%), the fixed effect model can be used for analysis, and the results still showed that the lead therapy group could improve the 6 MWT level of patients.[MD = 100.36, 95%CI (86.7114.1), *P* < .00001], unchanged results, low sensitivity, and relatively stable. For heterogeneity and the results showed heterogeneity (*P* < .00001, I^2^ = 94%), the random effect models were statistically significant, indicating that the Taijiquan group improved patients with the 6 MWT index. [MD ＝ 110.1, 95%C (21.61, 198.6), *P* = .01].

**Figure 7.**
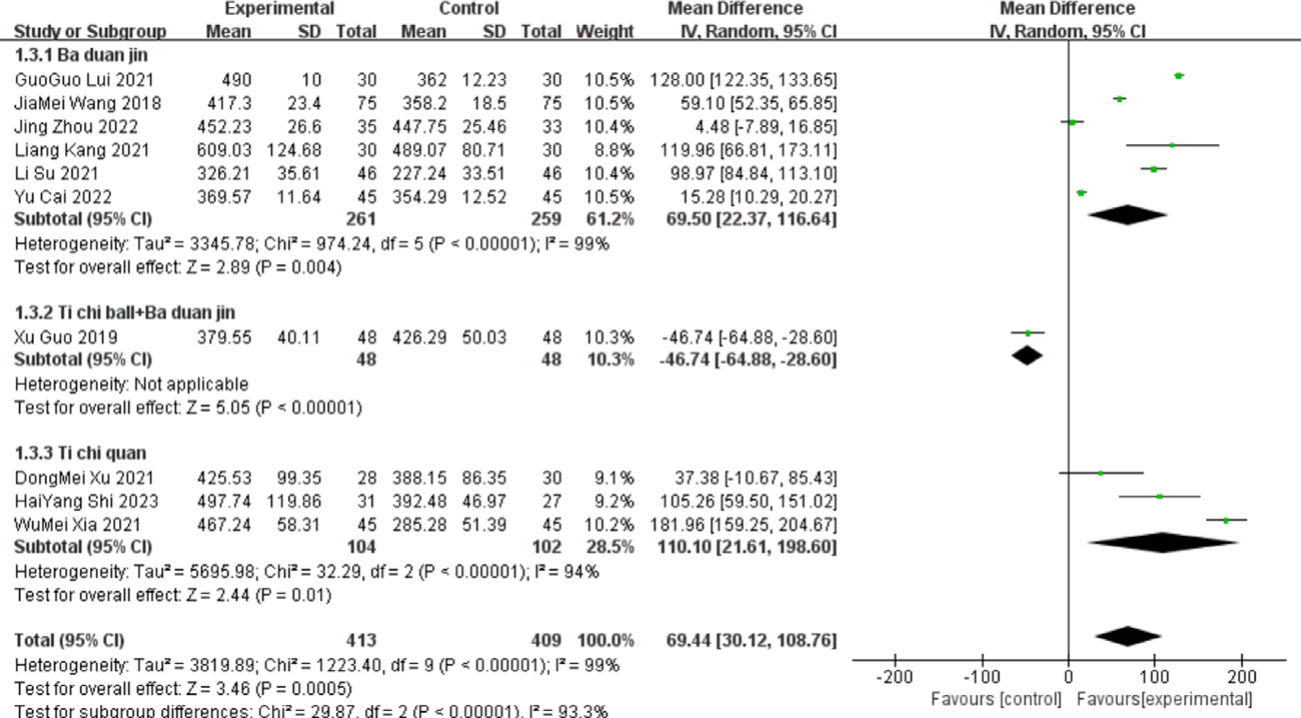
The meta-analysis results of group 6 MWT comparisons. 6 MWT = 6-minute walking distance.

**Figure 8.**
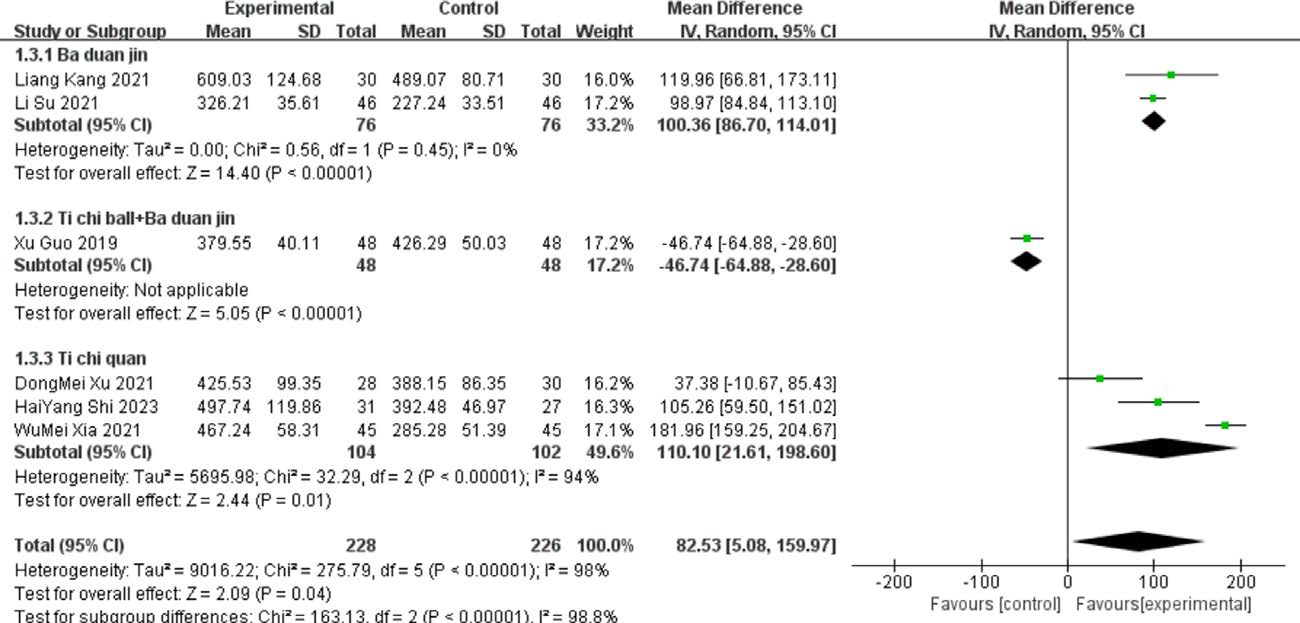
The meta-analysis results of 2 group 6 MWT comparisons after sensitivity analysis. 6 MWT = 6-minute walking distance.

As Tai Chi only combined the effect sizes of the 3 studies, the deletion heterogeneity did not change significantly. The^[25]^ course of Yu Dongmei was 4 months, the^[24]^ course of Shi Haiyang^[24]^ was 12 weeks, and the^[18]^ course of Xia Fumei was 8 weeks. The difference in treatment course may be the source of heterogeneity. Only one study was included in the Taiji ball + Baduanjin group; therefore, the heterogeneity test was not conducted. A Meta-analysis using a random effects model for the ten included studies showed that the lead group improved the patient 6 MWT index compared with the control group. [MD ＝ 69.44, 95%CI (30.12, 108.76), *P* < .000 01]. See Figures 7 and 8 for details.

#### 3.4.3. SF-36.

The SF-36 includes: physiological function (PF: physical function), physiological functions (RP: RolePhysical), physical pain (BP: bodily pain), general health (GH: General Health), vitality (VT: Vitalitv), social function (SF: SocialFunctioning), emotional function (RE: RoleEmotiona), and mental health (MHMentalHealth). Health change (HT: ReportHealthTransition). Six studies ^[11–12,16–17,24,31]^ reported the SF-36 scale, divided into 8 subgroups according to different test dimensions, and all were meta-analyzed using random-effect models. PF group: After the heterogeneity test for the 6 included studies, the results (*P* = .0005, I^2^ = 78%) and the lead group improved the PF compared with the control group, with a statistically significant difference of 0. [MD = 8.7, 95%CI (5.93, 11.62), *P* < .00001]; after the sensitivity analysis (*P* = .19, I^2^ = 35%). RP group: Six included studies passed the heterogeneity test and the results showed heterogeneity (*P* < .00001, I^2^ = 96%). The lead group showed improved RP compared with the control group, but the difference was statistically significant (MD = 13.2, 95%CI (6.07, 20.34), *P* = .0003). Through sensitivity analysis, heterogeneity decreased after excluding 3 studies^[17,24,31]^ (*P* = .93, I^2^ = 0%). Since the course of^[17,31]^ in 2 studies was 2 weeks and^[24]^ for 12 weeks, the difference in treatment courses was considered as the source of heterogeneity. BP group: For the 6 included studies, the heterogeneity test showed homogeneity (*P* < .00001, I^2^ = 90%), and the lead group showed improved BP compared to the control group [MD = 11.57, 95%CI (5.72, 17.42), *P* = .0001]. Through sensitivity analysis, heterogeneity decreased after excluding 2 studies^[17,24]^ (*P* = .37, I^2^ = 5%). Since the course of^[24]^ in this study was 12 weeks and that in another study^[17]^ was 2 weeks, the difference in the duration was considered as the source of heterogeneity. GH group: For the 5 included studies, through the heterogeneity test (*P* < .00001, I^2^ = 91%), the lead group showed improved GH compared with the control group, but the difference was not statistically significant (MD = 11, 95%CI (5.96, 16.05), *P* < .0001). Through sensitivity analysis, heterogeneity decreased after excluding 3 studies^[11,16,24]^ (*P* = 50.58, I^2^ = 0%). Since the^[11,24]^ course of this study was 12 weeks and the^[16]^ course was 12 weeks, the difference in courses was considered the source of heterogeneity. VT group: After the heterogeneity test, the 6 included studies showed significant heterogeneity (*P* = .0002, I^2^ = 79%), and the lead group showed significantly increased VT over the control group (MD = 10.67, 95%CI (7.47, 13.87), *P* < .000 1]. Sensitivity analysis revealed that the heterogeneity decreased (*P* = .57, I^2^ = 0%). Since the^[11]^ course of the study was 12 weeks and the^[16]^ course was 2 months, the difference in treatment course was considered as the source of heterogeneity.

**Figure 9.**
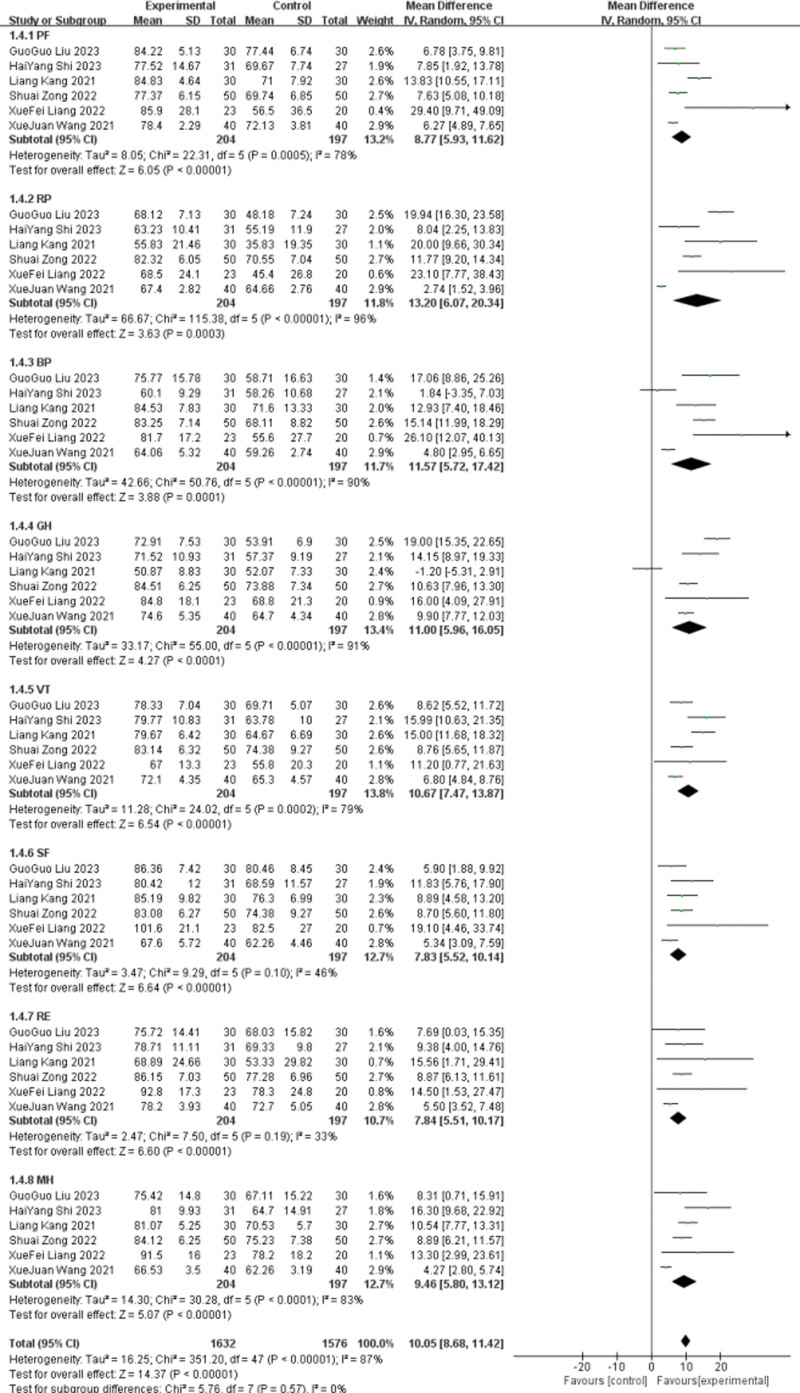
The meta-analysis results comparing group SF-36. SF-36 = quality of life.

**Figure 10.**
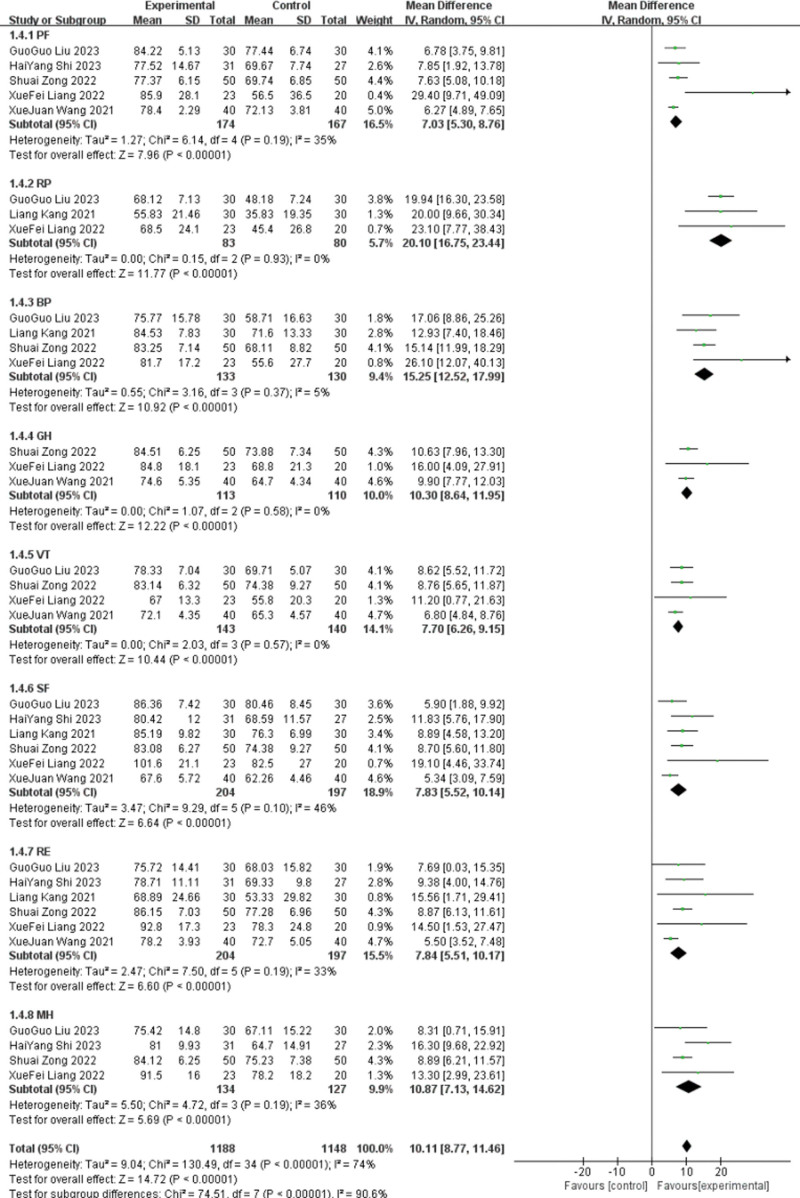
The meta-analysis results of 2-group SF-36 comparisons after sensitivity analysis. SF-36 = quality of life.

SF group: For the 5 included studies, the heterogeneity test showed homogeneity (*P* = .01, I^2^ = 46%), and the lead group showed improved SF compared to the control group [MD = 7.83, 95%CI (5.52, 10.14), *P* < .000 01]. RE group: The 6 included studies showed good homogeneity (*P* = .19, I^2^ = 33%), and the lead group showed improved RE compared with the control group, but the difference was statistically significant [MD = 7.84, 95%CI (5.51, 10.17), *P* < .00001]. MH group: Five included studies passed the heterogeneity test and showed heterogeneity (*P* < .0001, I^2^ = 83%), and the lead group showed improved MH compared with the control group (MD = 9.46, 95%CI [5.8, 13.12], *P* < .00001). Sensitivity analysis revealed that the heterogeneity decreased (*P* = .19, I^2^ = 36%). Since the^[16]^ course of the study lasted 2 months and the other study lasted^[17]^ for 2 weeks, the difference in the course was considered as the source of heterogeneity. See Figures 9 and 10 for details.

#### 3.4.4. SAQ.

The SAQ includes 5 dimensions: degree of somatic activity restriction (physical limitation, PL), stable state of angina pectoris (anginal stability, AS), frequency of angina attacks (anginal frequency, AF), treatment satisfaction (treatment satisfaction, TS), and awareness of the disease (disease perception, DS). Four studies^[16,22–23,28]^ reported on the SAQ, performing subgroup analyses with different dimensions, using random-effects models. PL integration group: The heterogeneity test of 4 studies showed heterogeneity (*P* = .11, I^2^ = 51%), and the guidance group showed PL statistically significant (MD = 6.26, 95%CI (2, 9.81), *P* = .0005). After sensitivity analysis, heterogeneity decreased (*P* = .3, I^2^ = 8%). For^[22]^ 2 months and 1 month, the difference in^[23]^ was considered as the source of heterogeneity. AS integral group: The 4 included studies showed homogeneity (*P* = .84, I^2^ = 0%), and the AS compared with the control group was statistically significant (MD = 10.14, 95%CI [6.51, 13.77], *P* < .00001).AF integral group showed heterogeneity (*P* = .04, I^2^ = 63%), and the lead group was statistically significant (MD = 5.79, 95%CI [1.58, 10.01], *P* = .007). Through sensitivity analysis, the heterogeneity decreased (*P* = .19, I^2^ = 40%) after excluding the study^[23]^ for 1 month; therefore, the difference in treatment period was considered as the source of heterogeneity. TS integral group: The heterogeneity test showed homogeneity (*P* = .78, I^2^ = 0%), and the lead group improved TS, but the difference was not statistically significant (MD = 1.4, 95%CI (−0.84, 3.64), *P* = .22).

**Figure 11.**
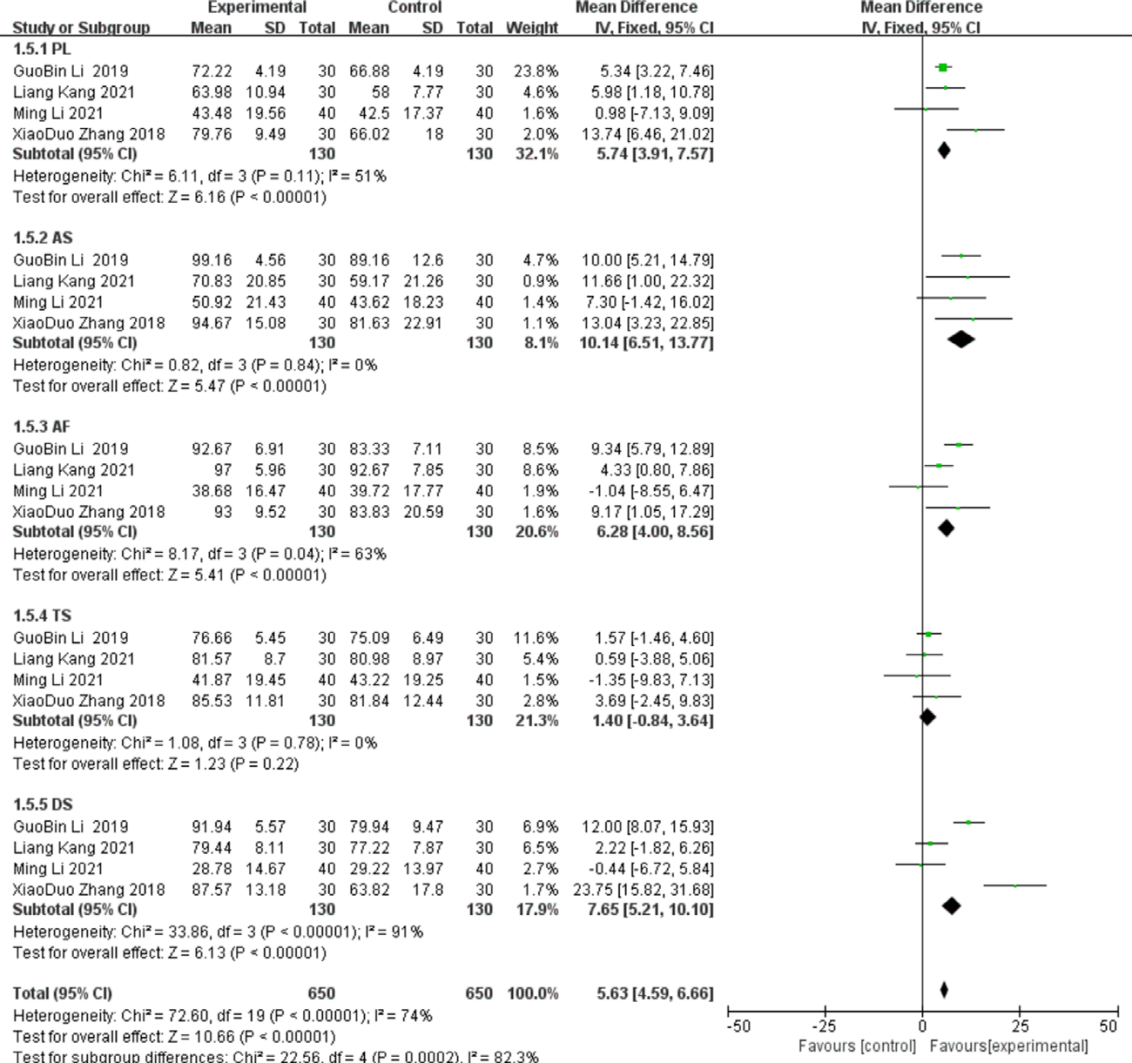
The results of the meta-analysis of group SAQ comparisons. SAQ = Seattle angina pectoris scale.

**Figure 12.**
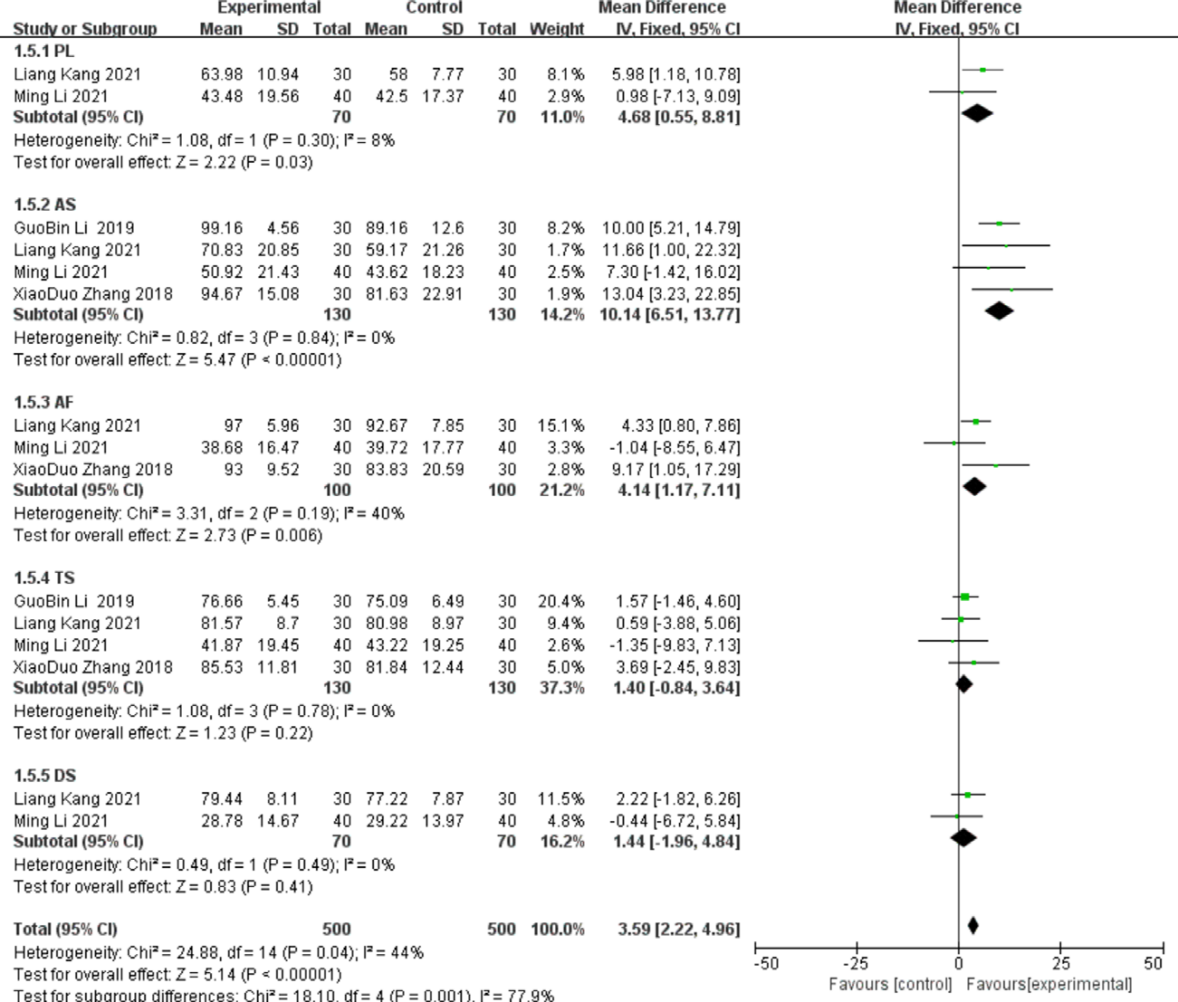
The meta-analysis results of 2-group SAQ comparisons after sensitivity analysis. SAQ = Seattle angina pectoris scale.

DS integration group: The heterogeneity test of 5 studies showed heterogeneity (*P* < .00001, I^2^ = 91%), and the lead group showed improved DS compared with the control group (MD = 9.05, 95%CI [0.36, 17.74], *P* = .04). Through sensitivity analysis, the heterogeneity decreased after excluding the study^[22,23]^ (*P* = .49, I^2^ = 0%). Since the^[22]^ course was 2 months and 1 month, the difference in^[23]^ course was considered as the source of heterogeneity. See Figures 11 and 12 for details.

#### 3.4.5. Incidence of adverse events, death rate, and readmission rate.

Six studies^[10,15,19,24,25,27]^ reported the adverse event rates. The heterogeneity test showed low heterogeneity among the studies (I^2^ = 0%), and a fixed-effects model was used. Meta-analysis showed the incidence of adverse events in the test group compared to the control group (RR = 0.17, 95%CI [0.1, 0.32], *P* < .00001).

Two^[21,25]^ studies reported on the incidence of death. The heterogeneity test showed low heterogeneity among the studies (I^2^ = 0%), and a fixed-effects model was used. Meta-analysis showed that the incidence of death decreased compared with that in the control group (RR = 0.29, 95%CI (0.1, 0.87), *P* < .00001).

**Figure 13.**
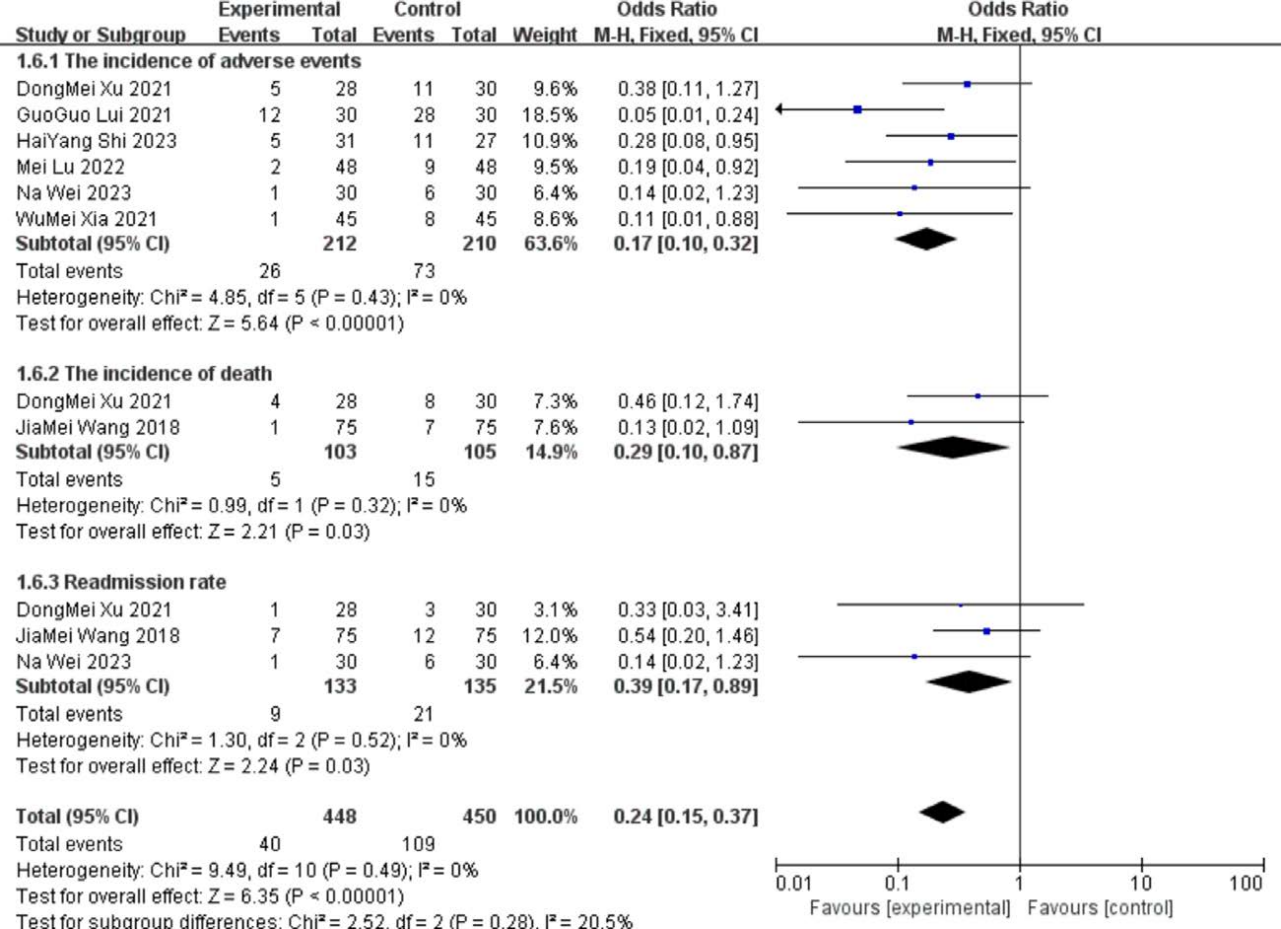
Metta-analysis of adverse event incidence, mortality and readmission.

Three^[10,21,25]^ studies reported the hospital readmission rates. The heterogeneity test showed low heterogeneity among the studies (I^2^ = 0%), and a fixed-effects model was used. The meta-analysis showed a lower readmission rate compared with the control group [RR = 0.39, 95%CI (0.17, 0.89), *P* < .00001]. See Figure 13.

#### 3.4.6. Publication bias.

**Figure 14.**
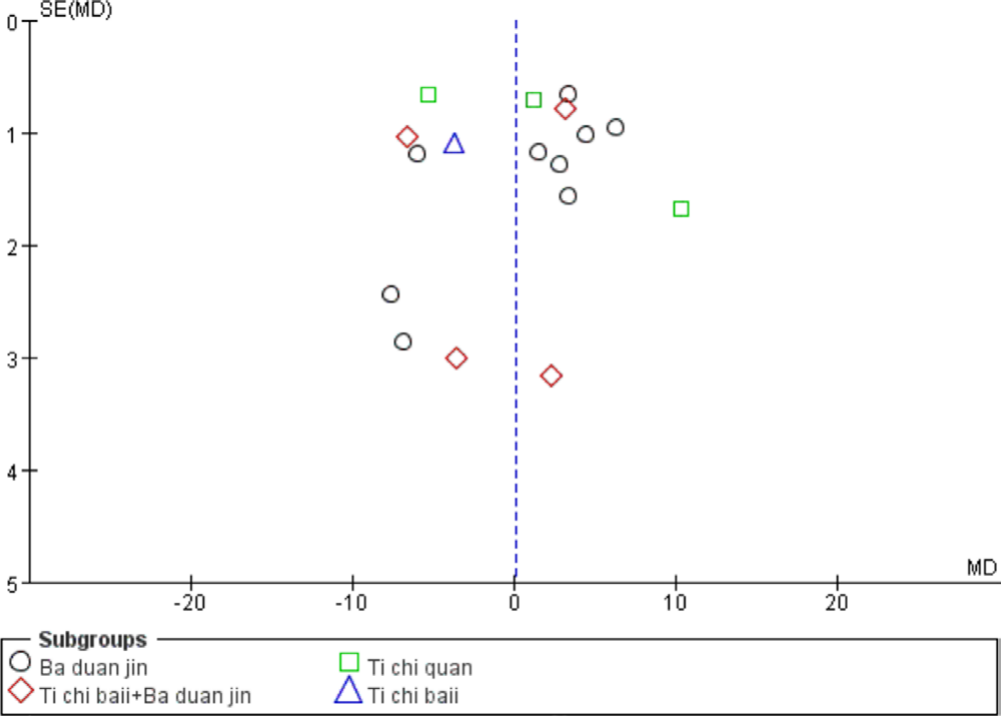
The LVEF funnel diagram. LVEF = left ventricular ejection fraction.

For the LVEF with the largest number of included articles published bias, the funnel diagram shows left and right asymmetry; the literature is centered on the vertical line distributed on both sides, most of which is concentrated in the upper part of the funnel. Left and right symmetry is poor, and the results show that there may be publication bias, which may be related to low literature quality factors (Fig. 14).

## 4. Discussion

TCM guiding therapy is based on the theory of TCM, combined with the overall concept of TCM, the theory of the meridians and the theory of the viscera, and integrates the movement of the operation of the viscera, and integrates the shape and spirit by “adjusting the body, interest and heart”.^[32–34]^ Can make the human body to achieve the effect of “Yin Pingyang secret” balance, because it can play a short-term treatment effect, and help to achieve the treatment and prevention of diseases, to achieve health care, improve the long-term effect of the overall quality of life of patients.^[35–36]^ There are many kinds of traditional TCM guidance, high selectivity, easy to operate, and less limited by time and site,^[35]^ Has obvious advantages over other modes of exercise. Currently, lead therapy is primarily used for the treatment of many diseases. Therefore, guidance therapy has a good research value for the clinical rehabilitation treatment effect after PCI for acute myocardial infarction. Indicators related to cardiac function are the most common objective indicators for evaluating the treatment effects of PCI after acute myocardial infarction. The SAQ scale is also an indicator of acute myocardial infarction. The SF-36 is an important indicator for evaluating the treatment of patients. Therefore, the above outcome indicators are of reference significance for evaluating the treatment effect and improving the quality of life after PCI for acute myocardial infarction.

The results showed that for the cardiac function-related indicators LVEF and LVEDV, the lead therapy and control groups significantly improved compared to the control group. For the 6 MWT index, the lead therapy group was also more effective than the control group. In the 8 dimensions of the SF-36, the lead treatment group scale score was higher than that of the control group, which improved the quality of life of the patients. However, the heterogeneity of these outcome indicators was high, and after sensitivity analysis, most of the causes of the heterogeneity were found. The SAQ scale and lead therapy group-related scores were also higher than those of the control group, with good homogeneity. Compared with the control group, the number and frequency of angina attacks were significantly reduced, maintaining a stable condition. The most obvious is that for the incidence of adverse events, fatality rate, and readmission rate, the incidence in the lead therapy group was lower than that in the control group, indicating that the safety, treatment effect, and stability of lead therapy have advantages compared with the control group. The advantage of lead therapy for patients after acute myocardial infarction PCI: Regular exercise of lead therapy will improve myocardial capillary density and blood perfusion, thus improving the contraction and relaxation ability of the heart, reducing myocardial ischemia, and improving the function of the vascular endothelium.^[37]^ Most Baduanjin and Taijiquan are medium- and low-intensity aerobic exercises. Whole body movements improve cardiopulmonary function, increase body endurance, exercise ability, balance of the body, and the patient ability to perform daily activities.^[38,39]^ Guiding therapy is a traditional work technique in ChinaStimulate Yang qi, the whole set of movements soft and slow, round live coherence, elastic combination, through the limb movement of the patient to warm heart Yang, relieve asthma, edema and other heart Yang symptoms, to achieve the purpose of improving the heart. At the same time, the movement emphasizes “interest adjustment.” In the process of the exercise, the deep breathing is combined to slow down the breathing rate, increase the movement range of the respiratory muscles, and adopt the exercise mode of blending Yin and Yang, vomiting, new forms and gods, which can cultivate one morality, and make the psychological state more stable and balanced.^[40–42]^ To improve the quality of life, and because of its high safety, not limited by the site equipment, low economic cost, and higher natural patient compliance, it can be vigorously promoted in clinical practice.

### 4.1. Limitations and perspectives of this study

The number of lead therapies included in this study is relatively small, including 13 Ba Duan Jin, 2 Tai Chi, 1 Tai Chi ball, 1 Tai Chi movement and 2 Tai Chi Jin; which may bias the research results. All documents were Chinese, and the research subjects were Chinese, which may have resulted in publication bias. The number of included documents was relatively small, including 8 badJin, 4 Taijiquan, 1 Shu Xin Yang Qigong, 1 Shaolin internal power, and 1 Yixin Cao. It is suggested that more high-quality research be conducted in the future. The interventions have not been standardized, and there is no unified standard for the intervention course, protocol, cycle, frequency, method, time, and intensity in the lead therapy literature in the included studies, resulting in heterogeneity among the literature. An expert consensus should be formed in the field of lead therapy treatment for acute myocardial infarction after PCI, and standardized guidelines for lead therapy treatment should be formulated to provide guidance for clinical practice. Common quality of the literature, including only 2 mentioned the blind and allocation scheme, the rest of the literature are not mentioned, may lead to selective bias, suggest that in the future clinical research, should pay attention to the blind and allocation scheme, ensure the completeness of the report results data, and improve the quality of RCTs.

## Author contributions

**Conceptualization:** Meixia Sun, He Zhuang.

**Data curation:** Meixia Sun, He Zhuang.

**Formal analysis:** Meixia Sun.

**Funding acquisition:** Meixia Sun, He Zhuang.

**Investigation:** Meixia Sun, He Yanwen.

**Methodology:** Meixia Sun, Yi Ding.

**Project administration:** Zhang Yukun.

**Resources:** Kang Chen.

**Validation:** He Zhuang.

**Visualization:** He Zhuang.

**Writing – original draft:** Yue Zhuo.
